# Food Policy Council Self-Assessment Tool: Development, Testing, and Results

**DOI:** 10.5888/pcd14.160281

**Published:** 2017-03-02

**Authors:** Larissa Calancie, Nicole E. Allen, Bryan J. Weiner, Shu Wen Ng, Dianne S. Ward, Alice Ammerman

**Affiliations:** 1 Center for Health Equity Research, Department of Social Medicine, School of Medicine, University of North Carolina at Chapel Hill, Chapel Hill, North Carolina; 2 Department of Psychology, University of Illinois at Urbana-Champaign, Champaign, Illinois; 3 Department of Global Health, School of Public Health, University of Washington, Seattle, Washington; 4 Carolina Population Center, University of North Carolina at Chapel Hill, Chapel Hill, North Carolina; 5 Department of Nutrition, Gillings School of Global Public Health, University of North Carolina at Chapel Hill, Chapel Hill, North Carolina; 6 Center for Health Promotion and Disease Prevention, University of North Carolina at Chapel Hill, 1700 Martin Luther King Jr. Blvd., CB# 7426, Chapel Hill, NC, 27599-7426.

## Abstract

A large number of food policy councils (FPCs) exist in the United States, Canada, and Tribal Nations (N = 278), yet there are no tools designed to measure their members’ perceptions of organizational capacity, social capital, and council effectiveness. Without such tools, it is challenging to determine best practices for FPCs and to measure change within and across councils over time. This study describes the development, testing, and findings from the Food Policy Council Self-Assessment Tool (FPC-SAT). The assessment measures council practices and council members’ perceptions of the following concepts: leadership, breadth of active membership, council climate, formality of council structure, knowledge sharing, relationships, member empowerment, community context, synergy, and impacts on the food system. All 278 FPCs listed on the Food Policy Network’s Online Directory were recruited to complete the FPC-SAT. Internal reliability (Cronbach’s α) and inter-rater reliability (AD, r_WG(J)_, ICC [intraclass correlations][1], ICC[2]) were calculated, and exploratory and a confirmatory factor analyses were conducted. Responses from 354 FPC members from 94 councils were used to test the assessment. Cronbach’s α ranged from 0.79 to 0.93 for the scales. FPC members reported the lowest mean scores on the breadth of active membership scale (2.49; standard deviation [SD], 0.62), indicating room for improvement, and highest on the leadership scale (3.45; SD, 0.45). The valid FPC-SAT can be used to identify FPC strengths and areas for improvement, measure differences across FPCs, and measure change in FPCs over time.

## Introduction

In a 2011 report by the Centers for Disease Control and Prevention (CDC), the first strategy recommended to increase fruit and vegetable consumption was to establish food policy councils (FPCs) as a way to improve the food environment at state and local levels (1). FPCs are organizations that bring together diverse members of the community to inform food policy and systems change and to coordinate or implement programs that aim to increase food access (2). In 2015, there were 278 FPCs in the United States, Canada, and Tribal Nations (3). FPCs are composed of representatives from many sectors of a local food system: producers, consumers, distributors, retailers, food processors, policy and decision makers, public health practitioners, food waste collectors, and hunger advocacy groups (4). FPCs’ organizational missions vary, but they often aim to inform changes that lead to increased access to nutritious foods for all members of the local food system through changes to agricultural, economic, environmental, and social programs and policies. FPCs may seek to inform policies and programs within organizations, or more broadly through municipal, county, or state-level policies (2).

Peer-reviewed literature, white papers, and guidance reports describe FPC activities, achievements, and challenges (2,5–7). Yet, few measurement tools exist to assess FPCs. Without such tools, best practices cannot be determined, and the mechanisms through which FPCs affect their food systems are difficult to explain. Measurement tools provide an opportunity to determine factors that differ across councils, determine what factors are associated with council effectiveness, and assess change within councils over time. Funders, researchers, FPCs, and the technical assistance groups that work with FPCs can all benefit from FPC-specific evaluation tools. Funders often require funding recipients to evaluate their work to determine the impact of their investments. Researchers also seek to evaluate practical approaches, such as FPCs, to complex, real-world problems. FPCs and the technical assistance groups that work with them can use measurement tools to conduct self-assessments to identify strengths and areas for improvement. No validated FPC-specific tools are available to evaluate councils.

The purpose of this study was to describe the development, testing, and findings from a self-assessment tool that measures FPC members’ perceptions of their council’s organizational capacity, social capital, and council effectiveness. The concepts measured by the self-assessment tool are informed by the literature on health-oriented community coalitions. The self-assessment tool can be used to guide FPC development, tailor capacity-building interventions for FPCs, and measure the internal functioning of FPCs.

## Conceptual Framework

**Figure 1:**
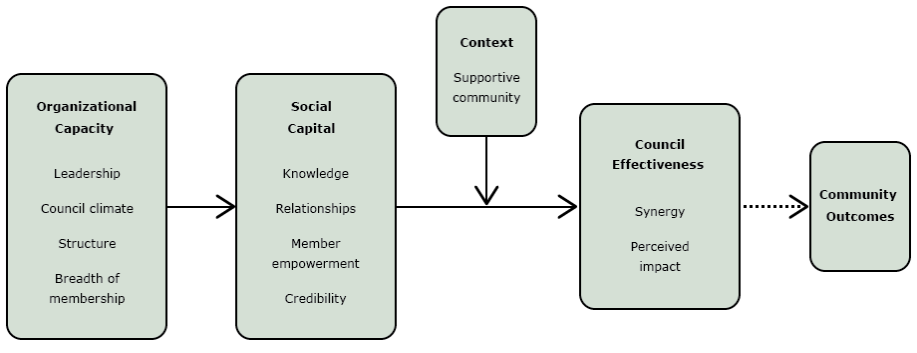
Food Policy Council Framework. Source: Allen NE, Javdani S, Lehrner AL, Walden AL. “Changing the text”: modeling council capacity to produce institutionalized change. Am J Community Psychol 2012;49(3-4):317–31.

We reviewed the literature on health-oriented community coalitions and FPCs to identify concepts to measure through the Food Policy Council Self-Assessment Tool (FPC-SAT). In reviewing the literature, we identified a parsimonious model explaining how community collaboratives influence institutionalized change in their communities (8). The concepts in the model aligned with the community coalition literature that we reviewed and depicted a set of relationships between concepts that we could test empirically (8–15). We adapted that model to create the FPC Framework (Figure 1) by including a credibility concept and by defining the outcome of interest as council effectiveness. Council effectiveness is defined as synergy, or the power to combine resources and perspectives to create new approaches to complex problems (13), and FPC council members’ perceptions of their councils’ impact on their food system. The concepts included in the FPC Framework are listed in Table 1.

**Table 1:** Concept Definitions for the Food Policy Council Framework^a^

Concepts	Definitions
**Organizational capacity**	
Leadership	Leaders promote an egalitarian or democratic environment, engaging participation from all members, valuing diversity, fair conflict management, articulating vision, and commitment to the group
Inclusive council climate	Shared power and decision making; shared mission; conflict resolution; sense of cohesion
Breadth of active membership	Range of stakeholders actively participating in council
Formality of council structure	Degree of structure guiding council practices and meetings
**Social capital and community context**	
Member empowerment	Degree to which members perceived being individually empowered to affect change (ie, to influence policy and practice in their home agencies and in the community) as a result of their participation in the council
Knowledge	Members are exposed to information about the food system and to each other’s activities related to the food system
Relationships	Connections between group members
Credibility of the council	Members’ perceptions about whether the community views the group as a trustworthy authority on food system related issues
Community context	Members’ perceptions of community members’ and decision-makers’ level of support for groups’ mission and activities
**Council effectiveness**	
Synergy	“The power to combine perspectives, resources, and skills of groups of people and organizations” (13, p. 183)
Perceived impact	Food council members’ perceptions of council-level accomplishments, or steps toward achieving the council’s goals

a Sources: Allen et al (8), Butterfoss and Kegler (9), Goodman et al (10), Granner and Sharpe (11), Kegler et al (12), Lasker et al (13), Roussos and Fawcett (14), Zakocs et al (15).

## Food Policy Council Self-Assessment Tool

### Item and scale generation

We drafted a preliminary set of questions for the FPC-SAT Tool based on past studies and the authors’ experience working with FPCs (2,4,8). Nine food council experts who either studied councils or provided technical assistance to councils provided feedback about the relevancy, clarity, and comprehensiveness of each question on the assessment (16). Food council experts were recruited through the Food Policy Council Working Group of the CDC-funded Nutrition and Obesity Policy Research and Evaluation Network (17) and through the North Carolina–based Community Food Strategies group (18). Experts included one who works with Tribal communities and who provided feedback that was specifically relevant to those populations. These experts provided input about the construct validity of the items (19). Two survey methodologists reviewed the questions and recommended simplifying the questions and providing additional question formats. The assessment was revised on the basis of feedback from content and methodology experts and entered into Qualtrics, an online survey platform.

### Cognitive response testing

A convenience sample of 4 food council leaders and members was recruited to complete cognitive response interviews. The participants represented a regional council in Washington State, a municipal council in New Jersey, and county-level councils in North Carolina and Kansas. Each participant was sent a link to the Qualtrics FPC-SAT immediately before a telephone interview. The interviewer conducted cognitive response interviews by asking the participants to read and answer the questions aloud to better understand how participants were interpreting the questions (20,21). Specific questions about response options, phrasing, and cognitive burden were also asked. The assessment was revised on the basis of these interviews.

### Reliability and validity

Cronbach’s α coefficients were calculated to determine the reliability of assessment items within each scale (22). Average deviation (AD_M(J)_) (23) and a within-group reliability index (r_wg(J)_) (24) were calculated to assess inter-rater agreement within FPCs. Interclass correlations (ICC[1], ICC[2]) were calculated from a one-way random effects analysis of variance (ANOVA) to quantify the effect of shared council membership on participants’ scale responses (25,26). ICC(1) estimates the amount of variation that can be explained by council membership and a high ICC(2) indicates that councils can be differentiated in terms of their members’ responses on a scale (25,27).

Exploratory factor analysis was used to examine whether items grouped together as predicted. Items with factor loadings of 0.3 or higher were grouped into that factor (19). Factors loadings were clarified by using an oblique rotation. Scales were created from the average of all the items measuring concept. “Not applicable” responses were considered missing data. Scales were grouped together into the following factors according to the FPC Framework: 1) Organizational capacity — leadership, breadth of active membership, formality of council structure, and inclusivity of council climate; 2) Social capital — knowledge, relationships, credibility, and member empowerment; and 3) Council effectiveness — synergy and perceived impact on the food system. Confirmatory factor analyses (CFA) were used to test whether scales correlated with these hypothesized factors. The CFA model was estimated by using maximum likelihood with missing values method. Bootstrapping was conducted and FPC members were clustered by their council identification during model estimation. CFA model fit was assessed using the Comparative Fit Index (CFI), the Tucker Lewis Index, and the Root Mean Square Error of Approximation (RMSEA) (28). Statistics were calculated by using STATA version 14.0 (StataCorp LLC) and the multilevel package version 2.5 in R (http://CRAN.R-project.org/package=multilevel). 

### Testing the tool

The 2015 online edition of the Food Policy Network FPC Directory was used to recruit members from all 278 FPCs in the United States, Canada, and Tribal Nations (3). Council members must have attended at least 2 meetings in the past year to be eligible to participate. Council contacts from the FPC Directory were recruited via email and asked to share the FPC-SAT link with their council members. Three reminder emails were sent to council contact persons, indicating how many council members had completed the assessment. Each participant could opt to receive a $5 Amazon e-gift card to incentivize individual members to participate. Councils with 8 or more participants received a feedback report summarizing their council’s aggregate responses as incentives for a high number of participants per council. Data were collected from July 2014 through October 2015. The institutional review board at the University of North Carolina, Chapel Hill, exempted this study.

Participant (N = 354) and council (N = 94) characteristics are listed in Table 2. Most participants were female (n = 240, 74%) and white (n = 271, 84%). Most had been members of their councils for 1 to 5 years (n = 237, 66%). The average age of council members was 6.27 years (standard deviation [SD], 5.10 y), and from 1 to 12 members per council completed the FPC-SAT.

**Table 2:** Participant (N = 354) and Council (N = 94) Characteristics, Food Policy Council Self-Assessment Tool

Characteristic	N (%)
**Participants**	
**Age, y**	
18–34	91 (27)
35–44	83 (25)
45–54	58 (18)
55–64	76 (23)
>65	20 (6)
**Sex**	
Male	86 (26)
Female	240 (74)
**Race/ethnicity **	
White	271 (84)
Hispanic	18 (6)
Black	13 (4)
American Indian or Aboriginal	4 (1)
Other	28 (8)
**Sector (participants could select >1)**	
Nonprofit	129 (36)
Agriculture	71 (20)
Community member	64 (18)
Education	62 (18)
Public health	60 (17)
Government	60 (17)
Other	40 (12)
Economic development	38 (11)
Academia	31 (9)
Poverty alleviation	26 (7)
Food security	26 (7)
Health care	18 (5)
Conservation	13 (4)
Faith	7 (2)
**Position **	
Leader (formal or informal)	51 (15)
Administration or staff (Secretary, Treasurer, Coordinator)	49 (14)
Chair of a working group or on a steering committee	77 (22)
Member	172 (49)
**Years as a member**	
<1	59 (17)
1 to <3	122 (34)
3 to <5	115 (32)
5 to <10	58 (16)
≥10	5 (1)
**General Council Characteristics**	
**Average council age in years (range 1–34)**	6.27 (5.10)
**Country **	
United States	82 (88)
Canada	11 (12)
Tribal nation (United States)	3 (3)
**Region **	
West	29 (32)
Midwest	16 (17)
South	23 (25)
Northeast	12 (13)
West (Canada)	2 (2)
Central (Canada)	8 (9)

Cognitive response participants said they understood the questions on the FPC-SAT and could easily answer most questions. Participants recommended some word changes (eg, be more specific about “experts,” remove or define “practitioners”) and adding several more response options to the “perceived impact” section. They also had several formatting suggestions: combining blocks of questions on a page so that the progress bar at the bottom of the survey moved more quickly, seeing a question stem at the top of a set of questions rather than re-reading the stem in a related set of questions, and being sure that the “submit” option is very clear on the last page. The 4 cognitive response participants recommended reducing the length if possible, but did not identify any particular questions or sections that seemed superfluous. One participant recommended providing an option to opt out of the gift card incentive because some government employees are not allowed to accept such incentives. We implemented all suggested changes.

Scale properties are listed on Table 3. From 267 to 353 participants selected a response other than “not applicable” to each item. Cronbach α ranged from 0.79 to 0.93, indicating high interrelatedness among items in each scale. Mean within-group agreement (r_wg(J)_) was above 0.70 for most scales, with the exception of relationships (r_wg(J) _= 0.69) and member empowerment (r_wg(J) _= 0.62), indicating that council members within councils generally agreed on their ratings for each scale (Table 3) (27). Mean AD_M(J)_ values below 0.67 indicate agreement among members within FPCs (Table 3) (27). All scales had mean AD_M(J)_ values of 0.67 or lower other than relationships (AD_M(J) _= 0.69) and member empowerment (AD_M(J) _= 0.67).

**Table 3:** Reliability and Validity of Items in the Food Policy Council Self-Assessment Tool Scale (N = 354)

Scale	No. of Items	Mean (SD)^a^	Cronbach α	r_WG(J)_	AD_M(J)_	ICC(1)	ICC(2)	*P *Value^b^
Leadership	7	3.45 (0.45)	0.88	0.96	0.36	0.05	0.17	.12
Breadth of active membership	6	2.49 (0.62)	0.80	0.88	0.51	0.28	0.58	<.001
Council structure	4	3.26 (0.60)	0.79	0.87	0.40	0.30	0.61	<.001
Council climate	5	3.03 (0.67)	0.84	0.81	0.52	0.17	0.42	<.001
Knowledge	6	2.96 (0.67)	0.86	0.72	0.62	0.04	0.13	.19
Relationships	5	2.86 (0.76)	0.91	0.69	0.69	0.11	0.31	.01
Credibility	3	2.58 (0.79)	0.92	0.74	0.53	0.21	0.48	<.001
Member empowerment	5	2.72 (0.79)	0.91	0.62	0.67	0.03	0.11	.24
Synergy	7	3.17 (0.51)	0.93	0.92	0.41	0.13	0.34	.01
Perceived impact	11	2.76 (0.51)	0.93	0.95	0.43	0.07	0.21	.08

Abbreviations: ICC, intraclass correlation; SD, standard deviation.

a Ratings are based on a scale of 1 to 4 where 1 is low and 4 is high.

b *P* values calculated by using analysis of variance.

The 1-way ANOVA models for each scale produced a range of ICC(1)s and ICC(2)s, indicating that council membership explained a portion of the variation in most but not all scales (Table 3). Council membership was a significant consideration for the following scales: breadth of active membership (*P* < .001), formality of council structure (<.001), inclusivity of council climate (*P* < .001), relationships (*P* = .01), credibility (*P* < .001), and synergy (*P* = .01).

Exploratory factor analysis indicated that the items grouped together within scales as expected with factor loadings of 0.3 or higher for each item. There was minimal cross loading (factor loadings of 0.3 or higher on more than 1 factor) between items from different scales, with the exception of some cross-loading between leadership and council climate items. Leadership sets the tone for whether a council has an inclusive climate and therefore may be difficult to parse in this assessment. The CFA was a good fit with the data (χ^2^ = 76.146, *df *= 32, *P* = <.001, CFI = 0.970, TLI = 0.958, RMSEA = 0.062, *p*-close = 0.121). These results indicate that the observed variables (the scales) were good measures of the hypothesized factors. The covariances between each factor ranged from 0.60 to 0.71 and were significant (*P* < .001), indicating that the factors are related yet distinct.

**Figure 2:**
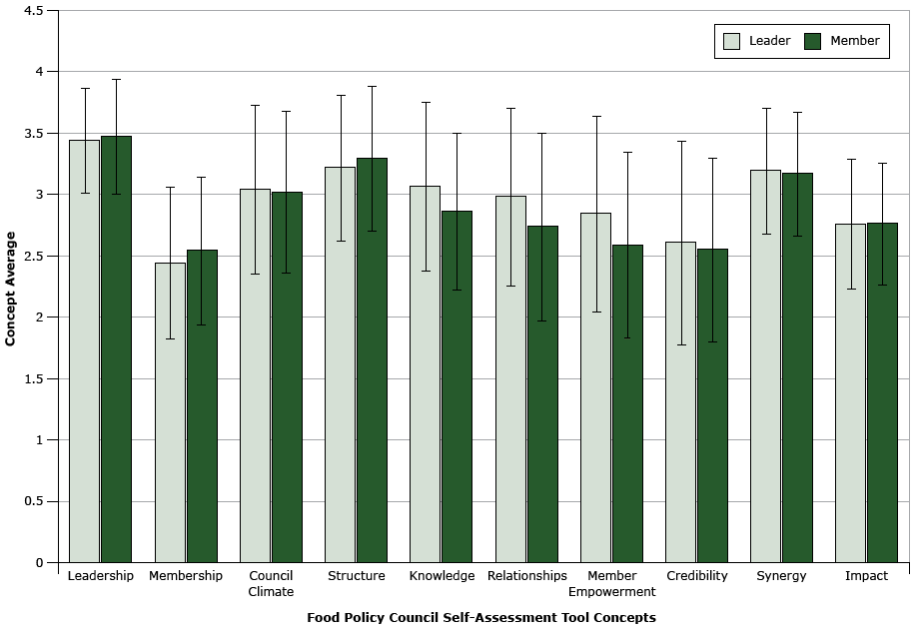
Concept means and standard deviations measured by the Food Policy Council Self-Assessment Tool (FPC-SAT) (range 1–4) for a sample (N = 354) of food policy council leaders and members. Leaders (formal or informal) (n = 51, 15%), administration or staff (secretary, coordinator) (n = 49, 14%), and working group chairs or members of steering committee (n = 77, 22%) were grouped together as leaders because of their additional investment in the councils. **Table:** 

We calculated concept averages from FPC leaders and members (Figure 2). Approximately half of participants reported “member” as their position within their councils (n = 172, 49%). The average concept scores were similar for the 2 groups. In the averaged combined scores of leaders and members, the leadership scale had the highest mean (mean, 3.45; SD, 0.45), followed by formality of council structure (mean, 3.25 SD, 0.60), and synergy (mean, 3.17; SD, 0.51). Breadth of active membership had the lowest scale average (mean, 2.49; SD, 0.62) followed by credibility (mean, 2.58; SD, 0.80, and impact (mean, 2.76; SD, 0.52) (Table 3). Table 4 shows the number of respondents, mean, and standard deviation for each of the 59 separate items in the scales.

Concept	Leader	Member
	Mean Value (Standard Deviation)	
Leadership	3.43 (0.43)	3.47 (0.47)
Membership	2.44 (0.62)	2.54 (0.61)
Council climate	3.04 (0.69)	3.02 (0.66)
Structure	3.21 (0.60)	3.29 (0.60)
Knowledge	3.06 (0.69)	2.86 (0.65)
Relationships	2.98 (0.73)	2.74 (0.77)
Member empowerment	2.84 (0.80)	2.59 (0.76)
Credibility	2.60 (0.84)	2.55 (0.75)
Synergy	3.19 (0.52)	3.17 (0.51)
Impact	2.76 (0.52)	2.76 (0.50)

**Table 4:** Respondents’ (N = 354) Ratings of Items on the Food Policy Council Self-Assessment Tool

Item	No. of Respondents	Rating^a^, Mean (SD)
**Leadership: In my opinion, the formal/informal leader(s) of our council . . .**		
Run effective meetings	352	3.33 (0.59)
Appear to devote adequate time to their position^b^	350	3.37 (0.58)
Are receptive to new ideas	351	3.54 (0.53)
Encourage all members to participate, not just loud or popular voices	352	3.49 (0.62)
Manage conflicts fairly	303	3.41 (0.59)
Encourage the council to move toward consensus on decisions^b^	345	3.50 (0.61)
Value diversity	349	3.56 (0.62)
**Breadth of active membership: In your opinion, to what extent . . .**		
Does your council include representatives from diverse sectors of the food system	354	2.94 (0.87)
Do the majority of the members in your council actively participate in the work of the council	352	2.41 (0.87)
Do you think your council has representation from the populations that council activities target	352	2.26 (0.90)
Does your council include a broad set of perspectives^b^	278	2.74 (0.81)
Is work shared evenly within the council	276	1.98 (0.77)
Do members actively get involved in the council	274	2.44 (0.76)
**Formality of council structure: In your opinion, how often does your council . . .**		
Seem well organized	354	3.13 (0.62)
Use written by-laws or guiding principles	342	2.97 (0.90)
Follow an agreed upon process for admitting new members into the council	323	3.34 (0.91)
Maintain records (eg, meeting minutes, time line of important events)	351	3.62 (0.62)
**Inclusivity of council climate: In your opinion, to what extent . . .**		
Is there a shared vision for the council among your councils' members	351	3.00 (0.84)
Do members in your council share power in decision-making	350	3.16 (0.84)
Is disagreement within your council resolved fairly	296	3.26 (0.80)
Do you think new members in your council feel welcome	342	3.07 (0.84)
Are you satisfied with the way your council functions	351	2.75 (0.94)
**Knowledge: To what extent has your participation in your council helped you learn about . . .**		
Policies that govern various aspects of the food system	352	3.01 (0.85)
Strategies to affect food system-related policies	351	2.87 (0.88)
The roles that other council members play in the food system	350	3.13 (0.84)
Food system-related needs or problems	353	3.11 (0.83)
The complexity of the food system^b^	350	3.17 (0.89)
The work of other food councils in your state or elsewhere^b^	352	2.49 (0.98)
**Relationships: To what extent has your participation in your council . . .**		
Improved your communication with other council members^b^	348	2.98 (0.83)
Improved your communication with the organizations that other council members belong to or represent	348	2.71 (0.92)
Helped you build trust with other council members	350	3.04 (0.84)
Helped you build trust with the organizations that other council members belong to or represent	345	2.81 (0.91)
Helped you coordinate efforts between your home organization and the organizations that other council members belong to or represent	328	2.77 (0.93)
**Credibility: To what extent . . .**		
Is your council viewed as a credible group within your community	342	2.60 (0.86)
Has your council established a positive reputation within your community	339	2.66 (0.85)
Is your council a group that the public views as a trustworthy source of information	332	2.55 (0.86)
**Member empowerment: To what extent has your participation in your council . . .**		
Helped you feel empowered to make food-related changes in your community or your home organization^b^	339	2.70 (0.93)
Led to opportunities to influence food system-related policies?	325	2.83 (0.96)
Led to opportunities to influence food system-related issues through programs or other non-policy efforts	337	2.81 (0.94)
Helped you become a champion for food-related issues in your community	334	2.64 (0.92)
Improved your confidence in your ability to make food-related suggestions to decision-makers in your home organizations	339	2.65 (0.87)
**Synergy: In my opinion, our council . . .**		
Has synergy, defined as “the power to combine the perspectives, resources, and skills of groups of people and organizations”	335	3.19 (0.60)
Develops creative solutions to food system-related issues^b^	330	2.99 (0.66)
Fosters holistic thinking related to the food system	335	3.18 (0.63)
Accomplishes goals that couldn't be achieved by a single organization^b^	330	3.23 (0.68)
Encourages practical solutions to food systems-related issues	333	3.18 (0.63)
Encourages comprehensive approaches to solving food system-related issues (eg, solutions that involve partners or that target multiple root causes of a problem)	335	3.17 (0.67)
Connects multiple food-related services, programs, or systems	336	3.26 (0.62)
**Impact: In my opinion, our council has . . .**		
Facilitated changes in policy or practice that will promote our council's mission	318	3.13 (0.62)
Stimulated policy change within my own organization	270	2.58 (0.73)
Increased access to healthy food in our community	318	3.02 (0.69)
Promoted social justice within the food system	324	3.03 (0.69)
Increased opportunities to purchase locally produced agricultural products	316	3.05 (0.71)
Increased the use of environmentally sustainable farming practices	301	2.63 (0.72)
Promoted occupational safety within the agricultural sector	269	2.22 (0.71)
Promoted humane treatment of animals within the agricultural sector	267	2.25 (0.67)
Facilitated distribution changes in our food system	288	2.46 (0.70)
Improved food safety practices in our community	308	2.84 (0.70)
Stimulated economic development in our community	304	2.77 (0.71)

a Ratings are based on a scale of 1 to 4 where 1 is low and 4 is high.

b Recommend removing these items from the abbreviated self-assessment tool.

The FPC-SAT asked participants to indicate their level of agreement with statements about their FPC’s impact on the food system in which they work (Table 4). Participants reported the areas of most impact as the following: facilitating changes in policy or practice that will promote our council's mission (mean, 3.13; SD, 0.62), increasing opportunities to purchase locally produced agricultural products (mean, 3.05; SD, 0.71), promoting social justice in the food system (mean, 3.03; SD, 0.69), and increasing access to healthy food in our community (mean, 3.02; SD, 0.70). Participants reported the lowest impact in the following areas: promoting occupational safety in the agricultural sector (mean, 2.22; SD, 0.71) and promoting humane treatment of animals in the agricultural sector (mean, 2.25; SD, 0.67). Additional research is needed to understand how FPCs prioritize which impact areas to pursue.

To make the tool more practical for repeated use by FPCs, we suggest abbreviating the tool. Table 4 shows suggested items to remove based on their effect on scale α values and their conceptual contribution to the concept scale. Table 5 shows the reliability of the abbreviated tool. The reliability measures are very similar in the original and abbreviated tool.

**Table 5:** Cronbach α of the Abbreviated Food Policy Council Self-Assessment Tool

Scale	No. of Items	Cronbach α
Leadership	5	0.85
Breadth of active membership	5	0.76
Formality of council structure	4	0.78
Inclusivity of council climate	5	0.84
Knowledge	4	0.85
Relationships	4	0.90
Member empowerment	4	0.90
Credibility	3	0.92
Synergy	5	0.87
Perceived impact	11	0.91
Total	50	NA

Abbreviation: NA, not applicable.

## Summary and Applications

This study described the development and testing of the first self-assessment tool adapted for FPCs to measure their members’ perceptions of their council’s organizational capacity, social capital, synergy, and impact on their food system. The FPC-SAT was adapted from Allen and colleague’s work and informed by literature on FPCs and community-based collaborations (4,8,15). Feedback from FPC members and experts was incorporated into the assessment. A final version of the assessment tool was tested with 354 council members from 94 councils in the United States, Canada, and Tribal Nations. Item reliability was high for all concept scales.

Researchers and practitioners can use the FPC-SAT to explore how FPCs function and why some may be more successful than others. Findings from the assessment tool may be useful in monitoring FPCs over time, especially before and after capacity-building interventions. The instrument could also reveal whether council members’ perceptions about their councils are similar or discordant and why that might be. Moreover, the FPC-SAT can help researchers identify the mechanism by which FPCs influence their food systems.

The FPC-SAT is a tool that council members and technical assistance groups can use to strengthen councils. In one study, authors interviewed and surveyed participants in an FPC and then provided feedback to the FPC regarding organizational capacity factors such as leadership (29). They reported an increase in council activity (eg, produced more media releases) following the provision of feedback (29). Training and technical assistance should be tailored to collaboratives’ needs (30), which can be illuminated by the FPC-SAT. For example, FPCs with high scores in certain areas on the FPC-SAT can focus improvement efforts on other areas that have more room for improvement. The FPC-SAT can also be used to measure the impact of training and technical assistance on specific items within scales or on scale averages.

In testing this assessment tool, we found several important patterns. Most FPC members are white women aged 35 to 65 years. A study of the relationship between community coalition factors and community impact found that greater racial diversity was associated with coalitions’ ability to change public policy and improve community prevention systems (31). FPCs may consider strategies for increasing diversity among their members. More research is needed to determine what member engagement strategies are effective at increasing member diversity. When looking at the mean scores for each concept scale, breadth of active membership, credibility, and impact had the lowest means. These are areas where councils may choose to direct more energy or seek external support such as training, technical support, and resources. Councils with low breadth of active membership scores, for example, may consider engaging potential members in a variety of settings. Councils can hold public forums where community members can provide input on the councils’ activities, or they can boost active participation by aligning council priorities with members’ goals and values.

Another interesting finding was the variability in inter-rater agreement and interclass correlation statistics among the scales. Inter-rater agreement and interclass correlations were both low for the member empowerment scale. Therefore, the member empowerment scale appears to capture perceptions that vary more among individuals, independent of their council affiliation, than the perceptions captured in the other scales. Researchers have found that community collaboratives can be empowering environments for their members but that members’ perceptions of empowerment can be influenced by other factors as well, such as the climate in their home organizations (32). Additional research into what factors explain variation in participants’ perceptions of empowerment in FPCs is warranted.

To our knowledge, this is the first study to recruit all members from all FPCs listed in the Food Policy Network’s FPC Directory (3). However, members from one-third of all councils listed in the directory submitted a survey. More than one member responded from only 35 councils, or 12% of total councils. FPC members who completed the assessment may be more invested in their councils or more motivated to see evaluation findings than those who did not complete the survey. The limited response rate may indicate that the survey was too long or that FPC members did not see the value in completing the assessment. The abbreviated tool may be more appealing to these members. Future work will test the use of the abbreviated FPC-SAT.

In conclusion, this study describes the Food Policy Council Self-Assessment Tool development, testing, and findings from council members in the United States, Canada, and Tribal Nations. Researchers, public health practitioners, and FPC members can use the tool to identify strengths and areas for improvement within councils and to measure change in these areas over time. Using the tool to understand council members’ perceptions of their councils’ organizational capacity, social capital, and council effectiveness can provide insight for researchers trying to determine how councils affect change in their food system. More research is needed to explore whether factors associated with FPC impact are not captured in the FPC-SAT. Empirical research is needed to test the relationships between the factors measured in the assessment to develop a theory or framework explaining how FPCs function.
